# An exploratory study on predicting HER2-positive expression status of breast cancer using ultrasound radiomics combined with machine learning models

**DOI:** 10.1371/journal.pone.0334909

**Published:** 2025-10-23

**Authors:** Xin-Ran Zhang, Sha-Sha Yuan, Jiao-Jiao Hu, Qing-Qing Chen, Yang-Jie Xiao, Ying-Fei Huang, Xiao-Qing Yu, Feng Lu, Yan Shen, Xiao-Hong Fu

**Affiliations:** 1 School of Gongli Hospital Medical Technology, University of Shanghai for Science and Technology, Shanghai, China; 2 Department of Ultrasound, Gongli Hospital, Shanghai Pudong New Area, Shanghai, China; 3 Department of Ultrasound, Shengjing Hospital, China Medical University, Shenyang, China; 4 School of Optical-Electrical and Computer Engineering, University of Shanghai for Science and Technology, Shanghai, China; 5 Center of Ultrasonography, Shuguang Hospital, Shanghai University of Traditional Chinese Medicine, Shanghai, China; Athens Medical Group, Psychiko Clinic, GREECE

## Abstract

**Objective:**

This study aimed to investigate the feasibility and potential value of predictive models for human epidermal growth factor receptor 2 (HER2)-positive status in breast cancer (BC) based on radiomics features from conventional ultrasound images and machine learning models.

**Methods:**

Ultrasound images of 437 patients with surgically and pathologically confirmed BC were retrospectively analyzed, including 144 HER2-positive and 293 HER2-negative cases, which were used as a training and validation dataset. Key features highly correlated with HER2-positive status were identified and selected using the least absolute shrinkage and selection operator (LASSO), t-test, and principal component analysis (PCA). After the selection of relevant features, the dataset was randomly split into five equal parts for five-fold cross-validation to identify the optimal machine learning method and hyperparameters. A predictive model was then developed based on ultrasound imaging and radiomics features. After feature selection and model development, an additional cohort of 88 patients from other hospitals was utilized as an external validation dataset. The model’s internal validation performance was assessed through receiver operating characteristic (ROC) curve analysis, and metrics including area under the curve (AUC), sensitivity, and specificity were calculated. The generalizability of the model was further evaluated using the external validation.

**Results:**

Five radiomics features were found to correlate with HER2-positive status in BC and used for model construction. Among the machine learning models generated, the best predictive model achieved area under the ROC curve values of 0.893 (95% confidence interval [CI], 0.860–0.920) in the training and validation dataset and 0.854 (95% CI, 0.775–0.927) in the external validation dataset.

**Conclusion:**

Machine learning models based on ultrasound radiomics features have potential clinical value for predicting HER2-positive status in BC.

## Introduction

Breast cancer (BC) is one of the most prevalent malignant tumors among women worldwide, and its incidence has continuously increased in recent decades [1]. Data from the World Health Organization (WHO) indicate that approximately 2.3 million new BC cases were diagnosed and approximately 670,000 BC-related deaths occurred globally in 2022, representing a serious threat to women’s health and survival [2]. Human epidermal growth factor receptor 2 (HER2) expression is found on 20–25% of BC cases, and HER2-positive tumors are known to be highly invasive, prone to brain metastasis, and associated with a poor prognosis. Fortunately, research has demonstrated that targeted drug therapy can significantly prolong patient survival and improve quality of life [3]. The main methods for detecting HER2 expression status are immunohistochemistry (IHC) analysis and fluorescence in situ hybridization (FISH) analysis of biopsy or surgically resected specimens. These methods are expensive, invasive, and time-consuming. Moreover, the incidence of HER2 expression heterogeneity in tumors is almost 40%, which means the detection of HER2 expression within only a small portion of tumor tissue may not reflect the overall expression status of the tumor [4–6]. In addition, changes in HER2 expression during neoadjuvant chemotherapy are observed in 20–40% of patients [7], further complicating analysis of HER2 expression status. Because intratumoral heterogeneity of HER2 expression was shown to be an independent influencing factor for inadequate response to neoadjuvant chemotherapy in HER2-positive patients [8], early detection of HER2 expression status is critical. However, the collection of multiple biopsy specimens from different tumor sites is challenging in clinical practice, and tumor heterogeneity introduces an unavoidable risk of bias. Therefore, a noninvasive and precise method for predicting the HER2 expression status of BC would offer a major advancement in the ability to provide individualized treatment for BC.

Ultrasound, as a safe, non-invasive, and convenient imaging technology, has been widely used in BC diagnosis and serves as an essential imaging modality for both preoperative screening and postoperative monitoring. In a preliminary study of the ultrasound characteristics of invasive metastatic BC, our group found that the combined application of multimodal ultrasound technology holds significant potential for differentiating benign and malignant lesions in breast BI-RADS category 4 nodes. This approach enhances the diagnostic accuracy for breast microcarcinomas. Additionally, we conducted an initial investigation into the ultrasound imaging characteristics of invasive metastases in BC and developed a preliminary model for their detection [9]. However, ultrasound examination is highly subjective and operator-dependent, limiting its ability to provide precise prediction. In recent years, as radiomics technology has rapidly developed [10], it has been widely applied in models for cancer differentiation. By extracting large-scale quantitative data from medical images, radiomics enables the analysis of tumor shape, intensity, texture, and intrinsic lesion characteristics. Furthermore, machine learning, as a powerful data analysis tool, can be applied to discover potential associations between imaging features and molecular features of tumors by learning from a large amount of medical image data, such as that produced by radiomics [11]. In the present study, we hypothesized that through the combination of radiomics and machine learning, the complex relationship between imaging features and HER2 expression status of BC can be comprehensively analyzed to establish accurate predictive models.

To test this hypothesis, in the present study, we constructed machine learning models to preoperatively predict HER2-positive status based on the ultrasound radiomics features of retrospectively analyzed BC cases. By comparing the predictive performance of different models, we aimed to identify the optimal model. Furthermore, we conducted external validation and SHapley Additive exPlanations (SHAP) analyses to assess the ability of the optimal prediction model to provide a theoretical basis for future clinical decision-making about treatment strategies.

## Materials and methods

### Patients

In this study, we retrospectively analyzed the data for BC patients admitted to Gongli Hospital of Shanghai Pudong New Area and Shuguang Hospital, Shanghai University of Traditional Chinese Medicine who were diagnosed via surgical pathology between January 2019 and December 2023. The data was accessed for research purposes on August 10, 2024 and August 18, 2024. These cases were used for the training and validation dataset. Additionally, BC patients admitted to Shengjing Hospital of China Medical University and diagnosed with BC via surgical pathology between January 2024 and August 2024 were included as an external validation dataset. The data was accessed for research purposes on September 5, 2024. The inclusion criteria were as follows: (1) complete IHC data; (2) clear and complete pre-operative breast ultrasound data; and (3) no fine needle aspiration biopsy performed prior to the breast ultrasound examination. Patients were excluded if they met any of the following exclusion criteria: (1) breast cancer intervention and treatment were administered prior to breast ultrasound screening; (2) the BC lesion exceeding the maximum single scanning range of the probe, making it too large for complete analysis in individual images. Cases were categorized as HER2-positive or HER2-negative according to the American Society of Clinical Oncology (ASCO)/College of American Pathologists (CAP) Clinical Practice Guidelines. HER2-positive status was defined as an IHC score of 3+ or an IHC score of 2+ with HER2 gene amplification [12]. A total of 525 patients were enrolled in the study, of which the training and validation dataset contained 437 patients, including 144 HER2-positive and 293 HER2-negative cases. The external validation dataset consisted of 88 patients, including 35 HER2-positive and 53 HER2-negative cases. This study was approved by the Ethics Committee of Gongli Hospital (Approval No: GLYYls2024−039) and adhered to the principles outlined in the Declaration of Helsinki. All participants provided written informed consent.

### Image acquisition and preprocessing

Ultrasound images in the training and validation datasets were acquired using the Philips EPIQ 7 system with an L12-5 linear array probe, operating within a frequency range of 5–12 MHz. Images in the external validation set were acquired using the Aixplorer system (SuperSonic Imagine, France) with an L14-5 linear array probe, operating within a frequency range of 5–14 MHz. Imaging parameters were uniformly set to a depth of 3–6 cm, a dynamic range of 60–65 dB, and an overall gain of 45–55 dB. These parameters were fine-tuned within the specified ranges based on breast thickness and image clarity. Additionally, time gain compensation was applied to ensure uniform brightness across the entire field of view. With patients positioned in a supine posture with arms raised above the head to ensure full exposure of both breasts. Ultrasound images of the primary breast lesion were acquired and analyzed to determine lesion location, morphology, size, internal echogenicity, posterior echogenicity, orientation, margins, calcifications, hypoechoic halo, burr sign presence, lobulation status, and blood flow characteristics. The acquired images were stored in DICOM format. Regions of interest (ROI) were manually delineated along the tumor margins by two independent senior ultrasonographers using the open-source software 3D Slicer 5.4.0 (https://www.slicer.org), following the double-blind principle. In cases of disagreement, consensus was reached through discussion, and the final segmentation was reviewed by an experienced senior physician. To assess the consistency of tumor segmentation, the Dice Similarity Coefficient (DSC) was first calculated. Subsequently, images from 30 randomly selected patients were re-annotated by a different physician, who had not been involved in the initial annotations, under single-blind conditions using the same method. The intraclass correlation coefficient (ICC) was then calculated to further analyze the consistency between the radiomics features derived from the second physician’s annotations and those from the initial physician’s annotations.

### Extraction and selection of imaging features

Ultrasound radiomics features were extracted using the 3D Slicer platform. The Least Absolute Shrinkage and Selection Operator (LASSO) regression algorithm was applied to select features with nonzero coefficients, and 8 features that exhibited statistically significant differences between HER2-positive and HER2-negative cases were identified using the t-test. Through dimensionality reduction using principal component analysis (PCA), the top radiomics features most strongly correlated with HER2-positive status were selected.

### Construction of machine learning models

The top-performing radiomics features were used to construct predictive models. The training and validation dataset was divided into five folds for training and internal validation. To mitigate the risk of overfitting, random oversampling was applied to balance the sample sizes of the two classes to 450:450. The model was then trained and validated based on the fold-specific case indices. External validation was performed using a dataset from another institution, where the trained model was applied to assess its performance. Ten commonly used machine learning classifiers were tested and compared. The performance metrics, including sensitivity, specificity, and area under the curve (AUC), were calculated through receiver operating characteristic (ROC) curve analysis for each machine learning model based on the training and validation dataset. The machine learning model that achieved the highest AUC was selected as the optimal ultrasound radiomics model for predicting the HER2-positive status of BC.

### Statistical analysis

SPSS 27.0 was used for statistical analysis. For normally distributed continuous variables, data are expressed as mean ± standard deviation (x ± s) and analyzed using the independent samples t-test. For non-normally distributed continuous variables, data are presented as median (Q1, Q3) and analyzed using the Mann-Whitney U test. SHAP analysis was employed to interpret the optimal model’s clinical value and to assess the contributions of individual radiomics features. The model’s generalizability was further evaluated by calculating performance metrics for the external validation dataset.

## Results

### Baseline characteristics of study participants

**Fig 1:**
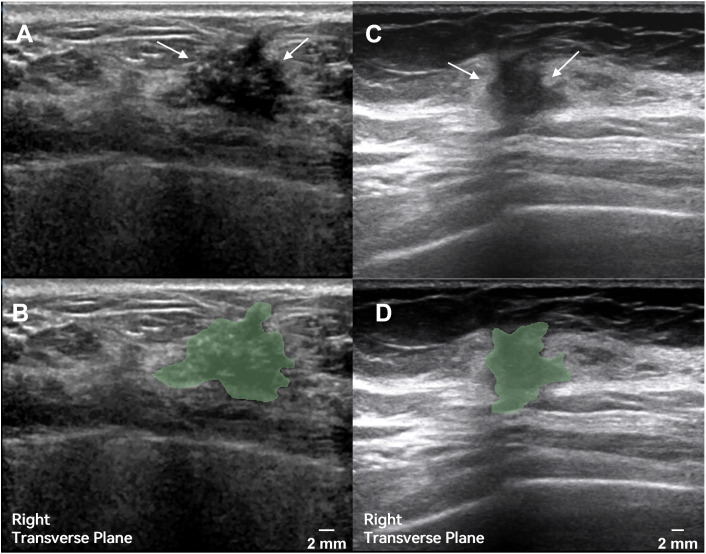
Representative ultrasound images of HER2-positive and HER2-negative tumors with ROIs drawn by ultrasonographers. **A.** Ultrasound image of a HER2-positive breast cancer patient. **B.** Corresponding ROI area for **A. C.** Ultrasound image of a HER2-negative breast cancer patient. **D.** Corresponding ROI area for **C.** Arrows in A and C indicate the tumor regions; the green areas in B and D represent tumor masking regions, annotated by two physicians in a double-blind manner. All images are cross-sectional views of the right breast, scale bar of 2 mm.

Data from three centers for a total of 525 BC patients were collected for this study. The training and validation dataset from the first and second centers included 437 cases, for which the mean age was 58.79 ± 13.48 years (range, 27–90 years), and the mean tumor size was 24.57 ± 13.38 mm (range, 3.4–135 mm). Of these cases, 144 were HER2-positive (age range, 27–88 years), and 293 were HER2-negative (age range, 27–90 years). The dataset from these two organizations was randomly divided equally into five groups for a five-fold cross-validation, and the average of the model’s metrics on the corresponding validation sets from the five cross-validations was used as the internal validation metrics. The external validation dataset consisted of 88 cases from the third center, with a mean age of 59.62 ± 12.84 years (range, 31–90 years) and a mean tumor size of 28.16 ± 14.79 mm (range, 5–80 mm). Of these cases, 35 were HER2-positive (age range, 31–88 years), and 53 were HER2-negative (age range, 32–90 years). The training and validation dataset was used for tuning as well as selecting hyperparameters, and the external validation dataset was used to test the performance of the model on different data. The training and validation dataset and the external validation dataset showed no differences in terms of age and tumor size (P > 0.05), However, statistically significant differences were detected between the HER2-positive and HER2-negative groups in terms of age and tumor size, with the exception of the tumor size of the patients in the external validation dataset (P < 0.05; Table 1). Using ROIs drawn on 2D ultrasound images from the BC patients in the training and validation dataset, a machine learning model for HER2 expression status was constructed for analysis and validation (Fig 1).

**Table 1:** Baseline clinical characteristics of BC patients.

	Training and validation dataset		P^*^	External validation dataset		P^*^
	HER2-positive	HER2-negative	HER2-positive	HER2-negative
Number	144	293		35	53	
Age (years)	54.86 ± 12.23	63.00 (51.00,70.00)	<0.001^*^	54.00 (50.00,62.00)	61.73 ± 13.31	0.018^*^
Size (mm)	24.00 (17.00,32.00)	21.00 (16.00,28.20)	0.033^*^	22.00 (18.00,36.00)	23.00 (19.50,38.50)	0.565

*: P value is considered significant.

test P < 0.05

### Selection of radiomic features predictive of HER2 expression status

**Fig 2:**
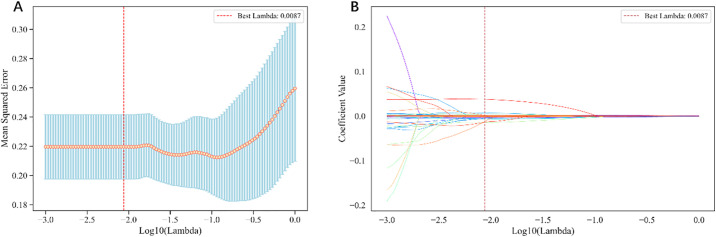
LASSO regression plot. **A.** Regularization parameter λ versus mean square error (MSE). Orange dots indicate the mean square error at different values of λ, and blue bars indicate the range of error fluctuations. The red dashed line marks the optimal λ value, which corresponds to the lowest mean square error when the model performs optimally. **B.** Trend of the coefficients for each feature as a function of the regularization parameter λ. Each curve represents the coefficient change for a feature under different values of λ. The red dashed line marks the optimal λ value when the model performs optimally.

**Fig 3:**
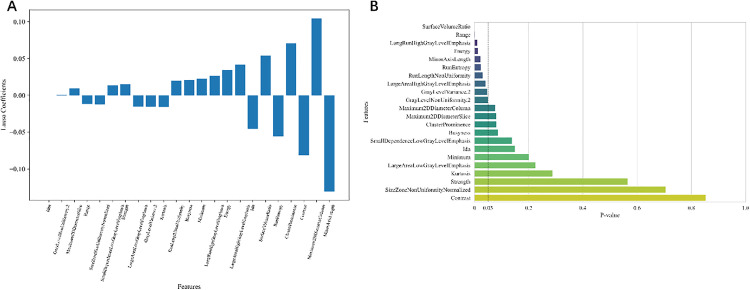
Feature selection and significance analysis. **A.** Feature significance bar graph: the length of the bar represents the significance of the feature. **B.** Feature significance analysis: the length of the bar represents the p-value of the feature, with shorter bars indicating a stronger significance between the feature and the target variable and longer bars indicating a weaker effect.

**Fig 4:**
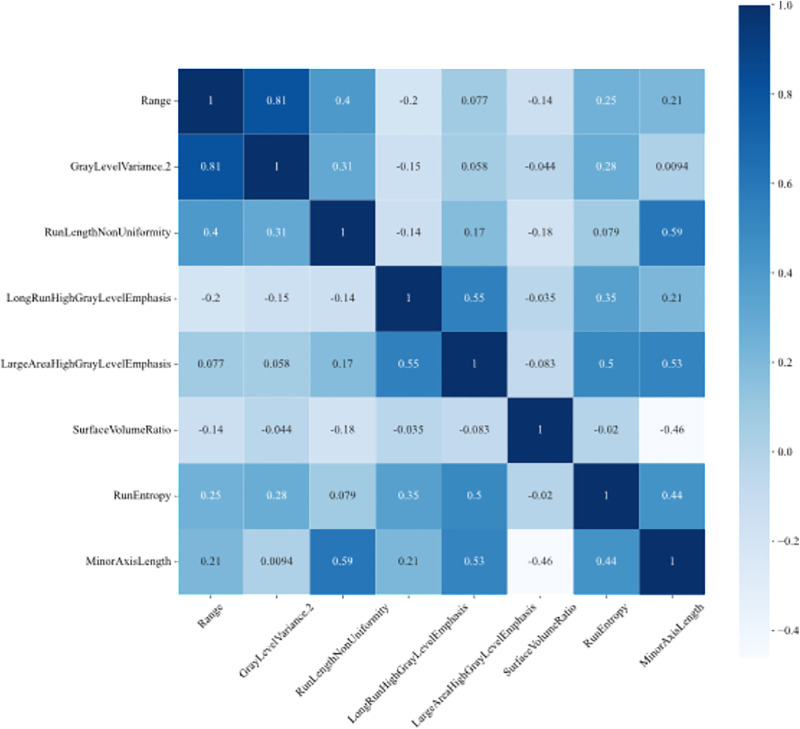
Feature correlation matrix. Pearson correlation coefficients between different features are presented, and the color shades indicate the strength of the correlation. Correlation coefficients close to 1 or −1 indicate strong positive or negative correlation, while those close to 0 indicate weak correlation.

**Fig 5:**
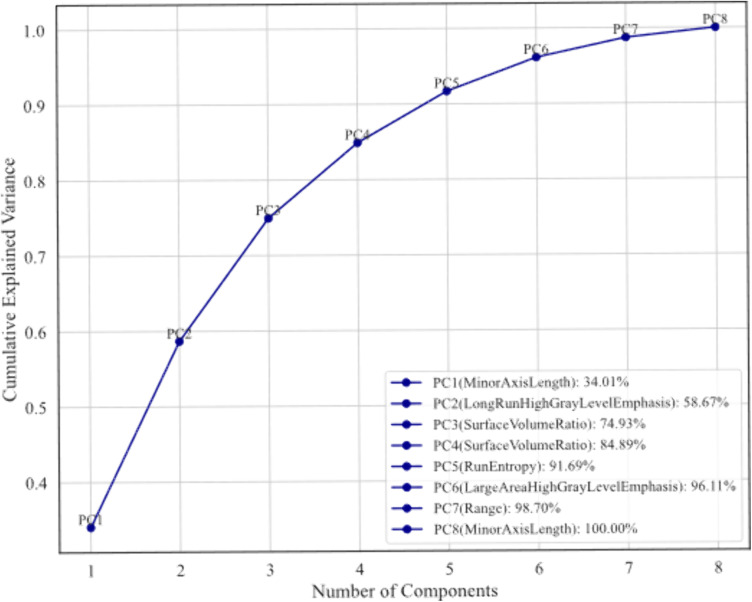
Cumulative explained variance plot from principal component analysis. This plot shows the variance contributed by different principal components (PCs) and the cumulative explained variance.

To assess inter-observer consistency, we first analyzed the consistency of tumor boundary delineation by different physicians. The results showed that the average DSC for the ROIs drawn by the two physicians was 0.910 (95% CI: 0.889–0.930). To further evaluate the consistency of radiomics features derived from the two physicians’ annotations, the intraclass correlation coefficient (ICC) was calculated. The ICC for the radiomics features showed good agreement between the two physicians (S1 Table). After the delineation was completed, we applied the LASSO regression algorithm to select features with significant predictive value for HER2 expression status in BC. This approach helped reduce overfitting and enhance feature selection, resulting in the identification of 22 features with nonzero regression coefficients from an initial set of 130 radiomics features (Fig 2). Using normalized regression coefficients and feature importance scores, we selected features with the highest predictive impact. These high-scoring features were incorporated into a final model for further evaluation (Fig 3). The t-test was employed to identify the following eight radiomics features with P-values <0.05 and their interrelationships, namely: range (RNG), gray level variance.2 (GLV.2), run length nonuniformity (RLN), long run high gray level emphasis (LRHGLE), large area high gray level emphasis (LAHGLE), surface to volume ratio (SVR), run entropy (RE), and minor axis length (MAL). To mitigate overfitting, we further analyzed the correlations among these features (Fig 4). Through correlation analysis, redundant features were identified to inform model optimization. Subsequently, to reduce model complexity and enhance interpretability and computational efficiency, we applied PCA to reduce feature dimensionality (Fig 5 and S2-S4 Tables). The balance between information retention and model simplification was determined using the cumulative explained variance (CEPV) 95% criterion, and the following top five contributing features were ultimately selected: LRHGLE, LAHGLE, SVR, RE, and MAL. They are all closely related to HER2-positive BC (S5 Table).

### Predictive performance of different machine learning models for HER2 expression status of BC

**Fig 6:**
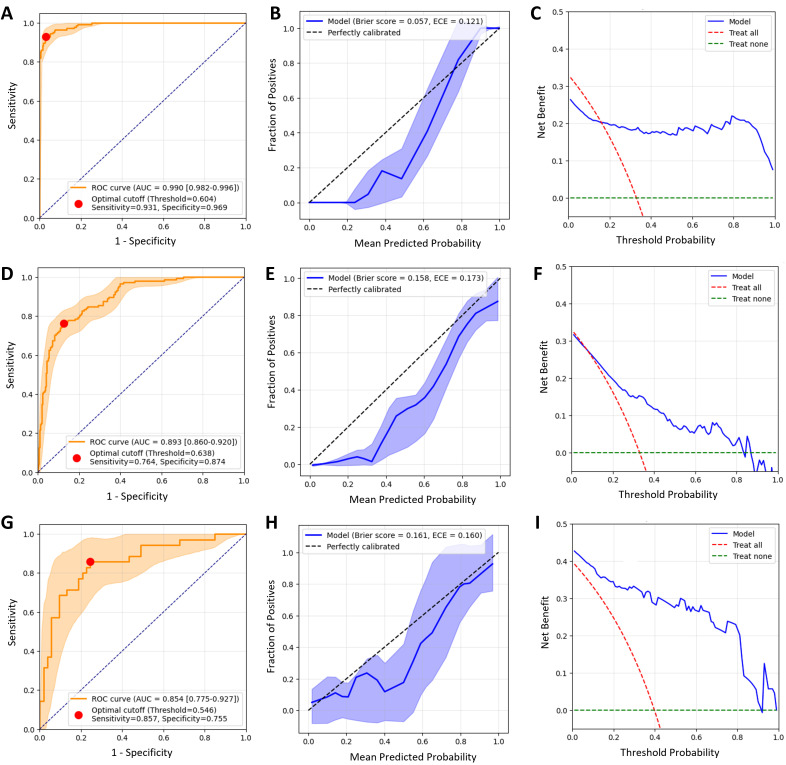
Performance metrics of the RF model. **A-C** show the ROC curve, calibration curve, and DCA curve for the training set; **D-F** show the ROC curve, calibration curve, and DCA curve for the internal validation set; **G-I** show the ROC curve, calibration curve, and DCA curve for the external validation set. The red dots on the ROC curves represent the points corresponding to the optimal threshold, and the confidence intervals for the curves were obtained using bootstrap (n = 2000).

The predictive performance of each model was evaluated through ROC curve analysis to calculate AUC, accuracy, sensitivity, specificity, and 95% confidence interval values. The RF-based model outperformed all other models. In the training and validation dataset and the external validation dataset, the AUC values for the RF-based model were 0.893 (95% CI: 0.860–0.920) and 0.854 (95% CI: 0.775–0.927), respectively, and these values were higher than those for the other models. The RF-based model also demonstrated superior accuracy, sensitivity, and specificity compared to most models, particularly in the external validation dataset, where its sensitivity and specificity were 0.829 and 0.736, respectively, demonstrating robust classification performance. The AUC confidence interval for the RF-based model was 0.775–0.927, indicating stable and reliable predictive performance. Therefore, the RF-based model was considered the model with the highest generalizability and predictive accuracy in this study. Thus, this model was selected for further analysis. To illustrate the model’s fit, we also included the metrics from the training set, including the confusion matrix, sensitivity, specificity, and AUC. (Tables 2 and 3, Fig 6, S6-S8 Tables).

**Table 2:** Performance metrics for the training and internal validation datasets.

Models	Datasets	Training and internal validation				
		ACC	SEN	SPC	Precision	AUC
KNN	Train	0.764(334/437) [0.723, 0.803]	0.646(93/144) [0.568, 0.722]	0.823(241/293) [0.780, 0.865]	0.641(93/145) [0.564, 0.719]	0.820 [0.778, 0.860]
	Val	0.696(304/437) [0.654, 0.737]	0.514(74/144) [0.437, 0.594]	0.785(230/293) [0.738, 0.831]	0.540(74/137) [0.455, 0.625]	0.689 [0.642,0.740]
LR	Train	0.531(232/437) [0.483, 0.581]	0.792(114/144) [0.725, 0.857]	0.403(118/293) [0.348, 0.459]	0.394(114/289) [0.337, 0.452]	0.589 [0.535, 0.646]
	Val	0.577(252/437) [0.529, 0.625]	0.722(104/144) [0.649, 0.797]	0.505(148/293) [0.447, 0.564]	0.418(104/149) [0.353, 0.483]	0.625 [0.571,0.680]
DT	Train	0.483(211/437) [0.435, 0.529]	0.903(130/144) [0.852, 0.949]	0.276(81/293) [0.226, 0.330]	0.380(130/342) [0.330, 0.432]	0.584 [0.526, 0.638]
	Val	0.682(298/437) [0.638, 0.725]	0.139(20/144) [0.083, 0.196]	0.949(278/293) [0.923, 0.973]	0.571(20/35) [0.400, 0.733]	0.642 [0.591,0.694]
SVM	Train	0.483(211/437) [0.435, 0.529]	0.965(139/144) [0.932, 0.993]	0.246(72/293) [0.198, 0.297]	0.386(139/360) [0.335, 0.435]	0.576 [0.520, 0.633]
	Val	0.570(249/437) [0.522, 0.620]	0.722(104/144) [0.651, 0.794]	0.495(145/293) [0.436, 0.554]	0.413(104/152) [0.351, 0.477]	0.639 [0.587,0.694]
XGB	Train	0.952(416/437) [0.931, 0.970]	0.993(143/144) [0.978, 1.000]	0.932(273/293) [0.902, 0.959]	0.877(143/163) [0.826, 0.927]	0.988 [0.976, 0.997]
	Val	0.822(359/437) [0.787, 0.856]	0.757(109/144) [0.684, 0.822]	0.853(250/293) [0.813, 0.893]	0.717(109/152) [0.644, 0.789]	0.845 [0.800,0.885]
RF	Train	0.957(418/437) [0.936, 0.975]	0.931(134/144) [0.887, 0.969]	0.969(284/293) [0.949, 0.987]	0.937(134/143) [0.896, 0.973]	0.990 [0.982, 0.996]
	Val	0.840(367/437) [0.803, 0.874]	0.792(114/144) [0.722, 0.854]	0.863(253/293) [0.822, 0.902]	0.740(114/154) [0.667, 0.810]	0.893 [0.860,0.920]
LDA	Train	0.572(250/437) [0.526, 0.622]	0.792(114/144) [0.725, 0.857]	0.464(136/293) [0.410, 0.523]	0.421(114/271) [0.361, 0.482]	0.584 [0.531, 0.641]
	Val	0.535(234/437) [0.490, 0.584]	0.722(104/144) [0.652, 0.798]	0.444(130/293) [0.384, 0.502]	0.390(104/267) [0.330, 0.451]	0.622 [0.567,0.678]
GBTR	Train	0.897(392/437) [0.867, 0.924]	0.840(121/144) [0.779, 0.898]	0.925(271/293) [0.894, 0.953]	0.846(121/143) [0.787, 0.903]	0.935 [0.905, 0.960]
	Val	0.794(347/437) [0.753, 0.831]	0.757(109/144) [0.683, 0.827]	0.812(238/293) [0.765, 0.855]	0.665(109/164) [0.587, 0.739]	0.819 [0.771,0.861]
MLP	Train	0.700(306/437) [0.657, 0.744]	0.500(72/144) [0.418, 0.586]	0.799(234/293) [0.753, 0.844]	0.550(72/131) [0.468, 0.639]	0.693 [0.644, 0.747]
	Val	0.648(283/437) [0.604, 0.691]	0.486(70/144) [0.406, 0.568]	0.727(213/293) [0.674, 0.776]	0.467(70/150) [0.386, 0.547]	0.659 [0.607,0.731]
LGBM	Train	0.968(423/437) [0.950, 0.984]	0.958(138/144) [0.922, 0.986]	0.973(285/293) [0.953, 0.990]	0.945(138/146) [0.906, 0.979]	0.988 [0.976, 0.997]
	Val	0.817(357/437) [0.780, 0.854]	0.861(124/144) [0.803, 0.912]	0.795(233/293) [0.747, 0.840]	0.674(124/184) [0.606, 0.743]	0.834 [0.786,0.872]

KNN: K-nearest neighbors; LR: logistic regression; DT: decision tree; SVM: support vector machine; XGB: XGBoost; RF: random forest; LDA: linear discriminant analysis; GBTR: gradient boosting decision tree; MLP: multi-layer perceptron; LGBM: lightGBM; ACC: accuracy; SEN: sensitivity; SPC: specificity; AUC: area under the curve; 95%CI: 95% confidence interval; Val: validation

Note. Data in parentheses are numerators/denominators; data in brackets are 95% CIs.

**Table 3:** Performance metrics for the external validation set.

Models	External validation				
	ACC	SEN	SPC	Precision	AUC
KNN	0.602(53/88) [0.500, 0.705]	0.743(26/35) [0.600, 0.882]	0.509(27/53) [0.373, 0.646]	0.500(26/52) [0.365, 0.634]	0.666 [0.555,0.774]
LR	0.670(59/88) [0.568, 0.773]	0.657(23/35) [0.487, 0.806]	0.679(36/53) [0.552, 0.800]	0.575(23/40) [0.421, 0.725]	0.755 [0.647,0.853]
DT	0.602(53/88) [0.500, 0.705]	0.943(33/35) [0.857, 1.000]	0.377(20/53) [0.250, 0.511]	0.500(33/66) [0.375, 0.617]	0.741 [0.635,0.840]
SVM	0.693(61/88) [0.591, 0.784]	0.657(23/35) [0.486, 0.806]	0.717(38/53) [0.582, 0.833]	0.605(23/38) [0.444, 0.750]	0.755 [0.633,0.860]
XGB	0.807(71/88) [0.716, 0.886]	0.800(28/35) [0.658, 0.926]	0.811(43/53) [0.692, 0.912]	0.737(28/38) [0.581, 0.872]	0.845 [0.752,0.924]
RF	0.773(68/88) [0.682, 0.852]	0.829(29/35) [0.704, 0.943]	0.736(39/53) [0.604, 0.848]	0.674(29/43) [0.525, 0.810]	0.854 [0.775,0.927]
LDA	0.682(60/88) [0.580, 0.773]	0.543(19/35) [0.371, 0.703]	0.774(41/53) [0.652, 0.885]	0.613(19/31) [0.433, 0.784]	0.747 [0.635,0.847]
GBTR	0.830(73/88) [0.739, 0.898]	0.829(29/35) [0.697, 0.943]	0.830(44/53) [0.717, 0.926]	0.763(29/38) [0.615, 0.889]	0.838 [0.745,0.919]
MLP	0.705(62/88) [0.602, 0.795]	0.771(27/35) [0.625, 0.903]	0.660(35/53) [0.519, 0.787]	0.600(27/45) [0.442, 0.744]	0.784 [0.678,0.874]
LGBM	0.807(71/88) [0.716, 0.886]	0.857(30/35) [0.735, 0.966]	0.774(41/53) [0.653, 0.878]	0.714(30/42) [0.564, 0.846]	0.826 [0.726,0.914]

KNN: K-nearest neighbors; LR: logistic regression; DT: decision tree; SVM: support vector machine; XGB: XGBoost; RF: random forest; LDA: linear discriminant analysis; GBTR: gradient boosting decision tree; MLP: multi-layer perceptron; LGBM: lightGBM; ACC: accuracy; SEN: sensitivity; SPC: specificity; AUC: area under the curve; 95%CI: 95% confidence interval; Val: validation

Note. Data in parentheses are numerators/denominators; data in brackets are 95% CIs.

### SHAP analysis of feature contributions to RF-based predictive model performance

**Fig 7:**
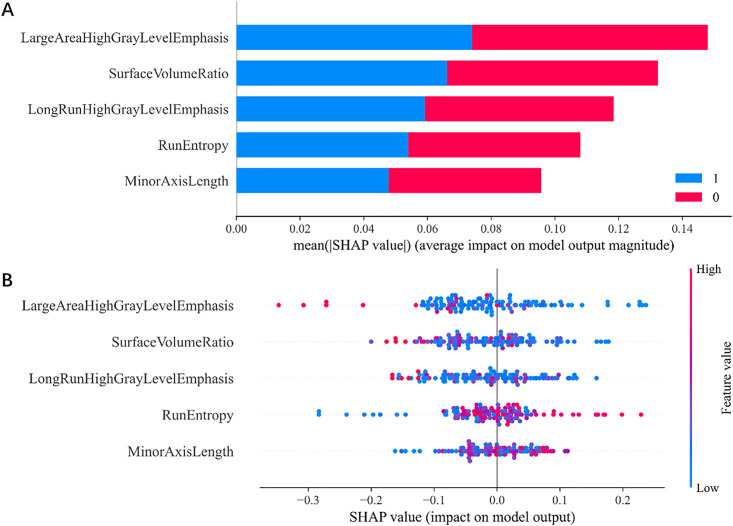
Feature importance vs. category distribution. **A.** Bar chart showing the average importance of each model feature under different categories, with blue and red representing the HER2-positive and HER2-negative categories, respectively. **B.** Scatterplot showing the distribution of each feature across samples, distinguishing between samples with different HER2 expression status by color.

According to the SHAP analysis results, the features LAHGLE and SVR made the greatest contributions to HER2 expression status prediction, with contribution values of 0.14 and 0.12, respectively (Fig 7A). The distribution of each feature across different categories was further analyzed. LAHGLE and SVR exhibited a distinct separation between the HER2-positive and HER2-negative categories, with blue and red scatter points distinctly representing differences between categories. Specifically, LAHGLE exhibited higher values in HER2-positive cases and lower values in HER2-negative cases (Fig 7B).

## Discussion

HER2 is a key component of the epidermal growth factor receptor (EGFR) pathway and plays a crucial role in regulating cell proliferation, differentiation, and survival. It is strongly associated with tumor initiation and progression, and has been identified as a marker of poor prognosis [13]. In the past, due to the lack of effective HER2-targeted treatments, HER2-positive BC represented one of the most aggressive subtypes of BC and had an extremely poor prognosis. However, the advent of effective anti-HER2 therapies has significantly improved the prognosis of patients with HER2-positive BC, with recent studies reporting a 5-year survival rate for patients with early-stage HER2-positive BC of 90% after treatment with a combination of chemotherapy, trastuzumab, and patulizumab. Moreover, the progression-free survival (PFS) and overall survival (OS) of patients with advanced-stage disease have also been markedly prolonged [14]. Therefore, early identification plays a critical role in guiding clinical treatment decisions for these patients.

Machine learning models have been shown to have high potential for use in predicting BC subtypes [15,16]. In this study, we identified the five most predictive radiomics features for HER2 expression status through radiomics analysis of ultrasound images from the training and validation dataset of the first and second centers. Based on these features, we developed 10 machine learning models for the prediction of HER2-positive status in BC. Our comparative analysis of model performance showed that the RF-based model exhibited superior predictive efficacy in the training and validation dataset and the external validation dataset, achieving AUC values of 0.893 and 0.854, respectively. Notably, the RF machine learning model integrates multiple decision trees for classification or regression tasks, providing greater accuracy and stability compared to a single decision tree. By randomly selecting features and samples, the RF model mitigates the overfitting issue associated with single decision trees, offering enhanced generalizability and high interpretability [17,18]. Our findings indicate that the construction of RF-based prediction models based on ultrasound radiomics features holds significant clinical value for predicting the HER2 expression status of BC. These findings are consistent with those of Ferre et al. [19], who developed a machine learning model based on ultrasound images that predicted HER2 expression status in BC with a sensitivity of 71.4%, a specificity of 71.6%, and an AUC of 0.778. Their study, however, was limited by a small sample size (only 88 cases) and the use of only three machine learning models.

In addition, previous studies also have utilized ultrasound parameters to predict BC subtypes. For example, Li et al. [20] investigated the association between molecular subtypes and imaging characteristics of BC to determine the ability of ultrasound features to predict different subtypes. Their results indicated that conventional ultrasound and ultrasonographic parameters exhibit some diagnostic value for identifying HER2-positive BC. However, ultrasound parameters are typically qualitative or semi-quantitative in nature, leading to subjective interpretation and limiting the amount of information that can be extracted from an ROI. In contrast, radiomics can extract high-dimensional textural and morphological features characteristic of tumor growth patterns, internal heterogeneity, and morphological classification, providing valuable insights for tumor diagnosis and prognosis prediction [21,22]. Machine learning algorithms enable the automated analysis of imaging features, mitigate human subjectivity, and offer significantly enhanced predictive performance and diagnostic efficiency. Machine learning has been applied for the differentiation between benign and malignant breast lesions, achieving promising results. However, the decision-making process of these models often lacks interpretability [23]. Therefore, the present study integrated radiomics with machine learning algorithms to develop a predictive model for the HER2 expression status of BC.

To mitigate the ‘black box’ effect of the radiomics-based model and support its credibility, we further analyzed and interpreted the prediction results through SHAP analysis, which accounts for interactions among individual variables and visually represents the positive and negative effects of variables using color [24–26]. According to the SHAP analysis results, the five included radiomics features contributed to the predictive performance of the model as ranked here in descending order: LAHGLE, SVR, LRHGLE, RE, and MAL. LAHGLE, LRHGLE, and RE are textural features, whereas SVR and MAL are morphological features. HER2-positive BCs tend to exhibit high LAHGLE, high SVR, high LRHGLE, high RE, and long MAL, indicating that these tumors exhibit greater textural complexity, a higher proportion of internal high-density regions (e.g., calcification, necrosis), and a more irregular morphological structure. These findings align with the aggressive biological behavior and high heterogeneity characteristic of this BC subtype.

Similar to our study, Cai et al. [27] applied a machine learning model for the differential diagnosis of triple-negative BC using two-dimensional ultrasound images. Their study also demonstrated that morphological and textural features play crucial roles in prediction performance, although external validation was not conducted. To mitigate the risk of overfitting, we used data from the third center for external validation in this study. In the external validation dataset, our RF-based model achieved an AUC of 0.854, with sensitivity and specificity of 0.829 and 0.736, respectively, demonstrating strong generalizability and achieving results consistent with those in the training and validation dataset, further validating the model’s reliability. However, despite the similarity with the internal validation set results, slight differences still exist, which may be attributed to variations in instrument models and physician techniques between different cohorts, potentially leading to some degree of fluctuation in the model’s stability.

However, the present study has several limitations. First, the sample size needs to be expanded to enhance the representativeness and generalizability of the dataset. Second, this study relied solely on two-dimensional ultrasound images, and the potential correlation between multimodal ultrasound imaging features and HER2-positive BC remains unexplored. Future studies can further validate such correlation by developing a more comprehensive multimodal imaging analysis model.

## Conclusion

In summary, the present study developed an integrated machine learning model based on ultrasound radiomics features that exhibited strong predictive performance for HER2 expression status in BC and good result interpretability and reliability, as demonstrated by SHAP analysis. The developed model holds promise as an auxiliary predictive tool for determining HER2-positive status in BC, potentially offering valuable insight for the clinical development of personalized treatment strategies.

## Supporting information

S1 Table ICC > 0.8. The intraclass correlation coefficient (ICC) was used to assess feature reliability, ranging from 0 to 1. Thresholds are commonly defined as: ICC < 0.5, poor; 0.5–0.75, moderate; 0.75–0.90, good; and >0.90, excellent. Based on the study design, the ICC(3,1) model (Two-way Mixed, Single Rater, Absolute Agreement) was applied, and features with ICC > 0.8 were retained for further analysis.
(DOCX)

S2 Table Principal component loadings matrix. (DOCX)

S3 Table Criteria for feature retention. (DOCX)

S4 Table Criteria for feature exclusion. (DOCX)

S5 Table Clinical interpretability of radiomic features. (DOCX)

S6 Table Confusion matrix of the training dataset. (DOCX)

S7 Table Confusion matrix of the internal validation dataset. (DOCX)

S8 Table Confusion matrix of the external validation dataset. Note: TP = True Positive; FP = False Positive; TN = True Negative; FN = False Negative.
(DOCX)
