# Supplementary material for: An exploratory study on predicting HER2-positive expression status of breast cancer using ultrasound radiomics combined with machine learning models
Source: PLoS One. 2025 Oct 23;20(10):e0334909. doi: 10.1371/journal.pone.0334909 (PMC12548876; doi:10.1371/journal.pone.0334909)
Supplement: S1 Table — The intraclass correlation coefficient (ICC) was used to assess feature reliability, ranging from 0 to 1. Thresholds are commonly defined as: ICC < 0.5, poor; 0.5–0.75, moderate; 0.75–0.90, good; and >0.90, excellent. Based on the study design, the ICC(3,1) model (Two-way Mixed, Single Rater, Absolute Agreement) was applied, and features with ICC > 0.8 were retained for further analysis. (DOCX) [file pone.0334909.s001.docx]

**S1 Table** ICC > 0.8

| Feature Labels | ICC |
| --- | --- |
| original_gldm_DependenceVariance | 0.819 |
| original_glrlm_LongRunHighGrayLevelEmphasis | 0.834 |
| original_glrlm_RunEntropy | 0.811 |
| original_glrlm_RunLengthNonUniformityNormalized | 0.811 |
| original_glszm_LargeAreaHighGrayLevelEmphasis | 0.850 |
| original_glszm_LowGrayLevelZoneEmphasis | 0.837 |
| original_glszm_SmallAreaLowGrayLevelEmphasis | 0.823 |
| original_shape_MinorAxisLength | 0.860 |
| original_shape_Sphericity | 0.895 |
| original_shape_SurfaceVolumeRatio | 0.944 |

The intraclass correlation coefficient (ICC) was used to assess feature reliability, ranging from 0 to 1. Thresholds are commonly defined as: ICC < 0.5, poor; 0.5–0.75, moderate; 0.75–0.90, good; and >0.90, excellent. Based on the study design, the ICC(3,1) model (Two-way Mixed, Single Rater, Absolute Agreement) was applied, and features with ICC > 0.8 were retained for further analysis.
