# Supplementary material for: An exploratory study on predicting HER2-positive expression status of breast cancer using ultrasound radiomics combined with machine learning models
Source: PLoS One. 2025 Oct 23;20(10):e0334909. doi: 10.1371/journal.pone.0334909 (PMC12548876; doi:10.1371/journal.pone.0334909)
Supplement: S2 Table — (DOCX) [file pone.0334909.s002.docx]

**S2 Table** Principal Component Loadings Matrix

| Feature Labels |  | PC1 | PC2 | PC3 | PC4 | PC5 | PC6 | PC7 | PC8 |
| --- | --- | --- | --- | --- | --- | --- | --- | --- | --- |
| MinorAxisLength |  | 0.483 | 0.171 | 0.388 | 0.160 | 0.218 | 0.001 | 0.364 | 0.618 |
| LongRunHighGrayLevelEmphasis |  | 0.171 | 0.531 | 0.242 | 0.171 | 0.530 | 0.550 | 0.126 | 0.027 |
| SurfaceVolumeRatio |  | 0.234 | 0.005 | 0.556 | 0.738 | 0.018 | 0.020 | 0.219 | 0.207 |
| RunEntropy |  | 0.397 | 0.196 | 0.379 | 0.059 | 0.710 | 0.237 | 0.168 | 0.262 |
| LargeAreaHighGrayLevelEmphasis |  | 0.394 | 0.389 | 0.161 | 0.126 | 0.256 | 0.731 | 0.167 | 0.157 |
| Range |  | 0.366 | 0.474 | 0.213 | 0.148 | 0.140 | 0.067 | 0.666 | 0.326 |
| GrayLevelVariance.2 |  | 0.310 | 0.469 | 0.378 | 0.193 | 0.189 | 0.027 | 0.455 | 0.510 |
| RunLengthNonUniformity |  | 0.375 | 0.231 | 0.353 | 0.567 | 0.216 | 0.319 | 0.311 | 0.339 |
