# Supplementary material for: An exploratory study on predicting HER2-positive expression status of breast cancer using ultrasound radiomics combined with machine learning models
Source: PLoS One. 2025 Oct 23;20(10):e0334909. doi: 10.1371/journal.pone.0334909 (PMC12548876; doi:10.1371/journal.pone.0334909)
Supplement: S3 Table — (DOCX) [file pone.0334909.s003.docx]

**S3 Table** Criteria for Feature Retention

| Feature Labels | Critical Loadings Locations | Retention Criteria |
| --- | --- | --- |
| MinorAxisLength | PC1(0.483), PC8(0.618) | Significant contribution in the morphological dimension (PC1), though exhibiting high loading on PC8, it is retained considering its clinical significance. |
| LongRunHighGrayLevelEmphasis | PC2(0.531),  PC5(0.530), PC6(0.550) | Multiple core components exhibit stable performance, reflecting high-dimensional long-range correlations. |
| SurfaceVolumeRatio | PC3(0.556), PC4(0.738) | Significant contributions in the spatial configuration dimensions (PC3, PC4), representing the tumor surface-to-volume ratio. |
| RunEntropy | PC1(0.397), PC5(0.710) | Significant performance in texture heterogeneity dimensions (PC1, PC5) Quantified uncertainty in operational length |
| LargeAreaHighGrayLevelEmphasis | PC1(0.394), PC6(0.731) | Significant contributions in the intensity-area composite dimension (PC1, PC6) reflect high signal intensity across large regions. |
