# Supplementary material for: An exploratory study on predicting HER2-positive expression status of breast cancer using ultrasound radiomics combined with machine learning models
Source: PLoS One. 2025 Oct 23;20(10):e0334909. doi: 10.1371/journal.pone.0334909 (PMC12548876; doi:10.1371/journal.pone.0334909)
Supplement: S4 Table — (DOCX) [file pone.0334909.s004.docx]

**S4 Table** Criteria for Feature Exclusion

| Feature Labels | Maximum Loadings | Exclusion Criteria |
| --- | --- | --- |
| Range | 0.666(PC7) | Significant only in high-order noise components (PC7), with a low cumulative proportion of explained variance. |
| GrayLevelVariance.2 | 0.510(PC8) | Only the higher-order component (PC8) demonstrated statistical significance, and it exhibited redundancy with the retained feature GrayLevelNonUniformityNormalized. |
| RunLengthNonUniformity | 0.567(PC4) | Although significant in PC4 loadings, it is highly correlated with RunEntropy (r > 0.8). Considering redundancy, it should be removed. |
