# Supplementary material for: An exploratory study on predicting HER2-positive expression status of breast cancer using ultrasound radiomics combined with machine learning models
Source: PLoS One. 2025 Oct 23;20(10):e0334909. doi: 10.1371/journal.pone.0334909 (PMC12548876; doi:10.1371/journal.pone.0334909)
Supplement: S5 Table — (DOCX) [file pone.0334909.s005.docx]

**S5 Table** Clinical Interpretability of Radiomic Features

| Feature Labels | Run Entropy (RE) | Minor Axis Length (MAL) | Surface Volume Ratio (SVR) | Long Run High Gray Level Emphasis (LRHGLE) | Large Area High Gray Level Emphasis (LAHGLE) |
| --- | --- | --- | --- | --- | --- |
| Feature Categories | Texture Features | Shape Features | Shape Features | Texture Features | Texture Features |
| Radiological Interpretation | This feature reflects the complexity of gray-level run distribution within the ROI. Higher values indicate more disordered gray-level patterns and greater unpredictability, suggesting increased tumor texture heterogeneity. | This feature represents the short-axis length of the tumor under elliptical fitting, reflecting its geometric scale in the shorter dimension. Higher values often indicate larger tumor volume or more complex boundary morphology. | This feature represents the ratio of a tumor's surface area to its volume. A higher value indicates a more complex tumor boundary and a larger surface area relative to its volume. | This feature indicates the significance of regions within the ROI that exhibit both a long gray-scale range and high gray-scale values. Higher values suggest a greater likelihood of extensive, contiguous high-gray-scale structures within the tumor. | This feature indicates the significance of large areas with high gray values within the ROI. A higher value signifies the presence of larger regions with elevated gray values within the lesion. |
| Biological Relevance | HER2 gene amplification leads to sustained activation of downstream pathways, placing tumor cells in a state of high proliferation. Concurrently, HER2 activation upregulates vascular endothelial growth factor (VEGF) expression, promoting angiogenesis. However, these newly formed vessels are often structurally abnormal and functionally deficient, resulting in uneven local blood supply.  As tumors rapidly expand, hypoxia and necrosis develop in certain regions, further exacerbating tissue complexity and heterogeneity. Consequently, elevated RE values may indicate highly complex internal texture in HER2+ breast cancer, reflecting its tissue heterogeneity and aggressive behavior. | HER2 belongs to the epidermal growth factor receptor (EGFR) family, which normally regulates cell growth and differentiation. Following HER2 gene amplification, its protein is overexpressed on the cell membrane, continuously activating downstream pathways. This leads to rapid cell proliferation and reduced apoptosis.  Concurrently, HER2 activation can induce epithelial-mesenchymal transition (EMT), enhancing cellular migration and infiltration capabilities. Consequently, elevated MAL values may indicate that HER2-positive breast cancer exhibits greater local infiltrative and invasive potential. | HER2 gene amplification and protein overexpression persistently activate the PI3K/AKT and MAPK signaling pathways, promoting rapid cell proliferation and inhibiting apoptosis. Concurrently, HER2 activation induces EMT, enhancing cell migration and infiltration capabilities, thereby facilitating tumor invasion beyond the original glandular structure into surrounding tissues.  Additionally, abnormal angiogenesis and stromal fibrosis increase tumor heterogeneity, further contributing to complex borders and irregular morphology. Consequently, elevated SVR values may indicate that HER2-positive breast cancer exhibits more aggressive and irregular biological behavior. | HER2-positive breast cancer cells exhibit rapid proliferation and significantly increased demand for oxygen and nutrients. However, their newly formed blood vessels are often functionally inadequate, struggling to meet these high metabolic demands. This leads to local hypoxia and necrosis.  Necrotic areas are often accompanied by cellular debris, protein deposition, and fibrous reactions. On ultrasound images, they may manifest as increased acoustic impedance differences and localized density irregularities, forming relatively large, contiguous high-gray-scale blocks. The LRHGLE feature quantifies the presence of such extensive high-gray-scale structures; thus, elevated LRHGLE values may indicate cellular enrichment within lesions, locally compacted structures, and heightened invasiveness. | HER2-positive breast cancer exhibits rapid proliferation leading to increased cell numbers and elevated tissue density. Concurrently, abnormal ductal architecture causes disorganized tissue arrangement. Enhanced matrix remodeling and fibrosis result in collagen deposition and stromal thickening. Collectively, these factors contribute to overall tumor tissue compaction, appearing on ultrasound images as extensive, continuous areas of high gray scale.  Due to the high gray-scale signal often exhibited by dense or fibrotic tissue, the LAHGLE feature quantifies this manifestation. Consequently, elevated LAHGLE values indicate that HER2-positive breast cancer possesses greater tissue density and invasiveness. |
