# Supplementary material for: An exploratory study on predicting HER2-positive expression status of breast cancer using ultrasound radiomics combined with machine learning models
Source: PLoS One. 2025 Oct 23;20(10):e0334909. doi: 10.1371/journal.pone.0334909 (PMC12548876; doi:10.1371/journal.pone.0334909)
Supplement: S6 Table — (DOCX) [file pone.0334909.s006.docx]

**S6 Table** Confusion Matrix of the Training Dataset

| Models | Training dataset | | | |
| --- | --- | --- | --- | --- |
|  | TP | FP | TN | FN |
| KNN | 93 | 52 | 241 | 51 |
| LR | 114 | 175 | 118 | 30 |
| DT | 130 | 212 | 81 | 14 |
| SVM | 139 | 221 | 72 | 5 |
| XGB | 143 | 20 | 273 | 1 |
| RF | 134 | 9 | 284 | 10 |
| LDA | 114 | 157 | 136 | 30 |
| GBTR | 121 | 22 | 271 | 23 |
| MLP | 72 | 59 | 234 | 72 |
| LGBM | 138 | 8 | 285 | 6 |

Note: TP = True Positive; FP = False Positive; TN = True Negative; FN = False Negative.
