# Supplementary material for: An exploratory study on predicting HER2-positive expression status of breast cancer using ultrasound radiomics combined with machine learning models
Source: PLoS One. 2025 Oct 23;20(10):e0334909. doi: 10.1371/journal.pone.0334909 (PMC12548876; doi:10.1371/journal.pone.0334909)
Supplement: S7 Table — (DOCX) [file pone.0334909.s007.docx]

**S7 Table** Confusion Matrix of the Internal Validation Dataset

| Models | Internal validation dataset | | | |
| --- | --- | --- | --- | --- |
|  | TP | FP | TN | FN |
| KNN | 74 | 63 | 230 | 70 |
| LR | 104 | 145 | 148 | 40 |
| DT | 20 | 15 | 278 | 124 |
| SVM | 104 | 148 | 145 | 40 |
| XGB | 109 | 43 | 250 | 35 |
| RF | 114 | 40 | 253 | 30 |
| LDA | 104 | 163 | 130 | 40 |
| GBTR | 109 | 55 | 238 | 35 |
| MLP | 70 | 80 | 213 | 74 |
| LGBM | 124 | 60 | 233 | 20 |

Note: TP = True Positive; FP = False Positive; TN = True Negative; FN = False Negative.
