# Supplementary material for: An exploratory study on predicting HER2-positive expression status of breast cancer using ultrasound radiomics combined with machine learning models
Source: PLoS One. 2025 Oct 23;20(10):e0334909. doi: 10.1371/journal.pone.0334909 (PMC12548876; doi:10.1371/journal.pone.0334909)
Supplement: S8 Table — Note: TP = True Positive; FP = False Positive; TN = True Negative; FN = False Negative. (DOCX) [file pone.0334909.s008.docx]

**S8 Table** Confusion Matrix of the External Validation Dataset

| Models | External validation dataset | | | |
| --- | --- | --- | --- | --- |
|  | TP | FP | TN | FN |
| KNN | 26 | 26 | 27 | 9 |
| LR | 23 | 17 | 36 | 12 |
| DT | 33 | 33 | 20 | 2 |
| SVM | 23 | 15 | 38 | 12 |
| XGB | 28 | 10 | 43 | 7 |
| RF | 29 | 14 | 39 | 6 |
| LDA | 19 | 12 | 41 | 16 |
| GBTR | 29 | 9 | 44 | 6 |
| MLP | 27 | 18 | 35 | 8 |
| LGBM | 30 | 12 | 41 | 5 |

Note: TP = True Positive; FP = False Positive; TN = True Negative; FN = False Negative.
